# Noiseless Variable-Pressure Neck Chamber Device to Assess the Carotid Baroreflex Function

**DOI:** 10.3389/fphys.2020.613311

**Published:** 2021-01-20

**Authors:** Alessandro Pinheiro, Lauro C. Vianna, Jake C. Carmo

**Affiliations:** ^1^Faculty of Technology, University of Brasília, Brasília, Brazil; ^2^Federal Institute of Education, Science, and Technology of Brasília, Brasília, Brazil; ^3^NeuroV̇AṠQ – Integrative Physiology Laboratory, Faculty of Physical Education, University of Brasília, Brasília, Brazil; ^4^Graduate Program in Medical Sciences, Faculty of Medicine, University of Brasília, Brasília, Brazil; ^5^Biological Signals Processing Laboratory, Faculty of Physical Education, University of Brasília, Brasília, Brazil

**Keywords:** blood pressure, baroreflex, neck suction, hypertensive stimulus, neck collar

## Abstract

**Background**: The blood pressure responses to baroreflex perturbations can be assessed only using the variable-pressure neck chamber technique. However, the application of this approach in hospital environments is limited owing to the loud noise emitted during its operation. This study was aimed at developing a noiseless neck suction chamber device (NCD) that could stimulate the baroreceptors located in the carotid sinus in humans.

**Methods**: A non-invasive device was developed to pressurize the carotid arteries externally. A microcontroller with a computer interface and neck chamber (3D-printed) was used. The anatomical neck chamber was fitted on six healthy, young, asymptomatic participants (five men; 32 ± 6 year), who were normotensive, nonsmoking, in sinus rhythm, free of known cardiovascular or metabolic diseases, and not consuming any acute or chronic medications. A suction of −60 mmHg was applied for 5 s, and the corresponding data were recorded. Before each study visit, the participants were instructed to abstain from caffeine, alcohol, and strenuous exercise for 12–24 h.

**Results**: In all the trials, a significant reflex bradycardia (−10 ± 2 bpm) and depressor response (−15 ± 4 mmHg) to neck suction were observed, consistent with the results in the literature. The neck chamber device operated noiselessly [sound pressure level (SPL) of 34.3 dB] compared to a regular vacuum-cleaner-based system (74.6 dB).

**Conclusion**: Using the proposed approach, consistent blood pressure and heart rate responses to carotid baroreflex hypertensive stimuli could be recorded, as in previous studies conducted using neck collar devices. Furthermore, the neck chamber device operated noiselessly and can thus be applied in hospital environments.

## Introduction

The arterial baroreflex system plays a pivotal role in the short-term regulation of blood pressure and cardiovascular variability (Eckberg and Sleight, 1992). Nevertheless, several factors (related to the sex, age, health, and environment of an individual; Cooper et al., 2007; Kim et al., 2011; Credeur et al., 2014; Kaufmann et al., 2020) may influence the gain and effectiveness of the baroreflex, along with the cardiovascular variability. Furthermore, many central neural structures help regulate the cardiovascular system and thus contribute to the integrity of the baroreflex (Chapleau et al., 1989). Notably, abnormalities in the arterial baroreflex function have been linked to a degradation of the cardiovascular variability, deterioration of the cardiovascular outcomes, and mortality in several diseases (La Rovere et al., 1998). Therefore, assessing the baroreflex function is of significance in both healthy and diseased individuals, especially in the context of the prognostic evaluation and assessment of the effect of the treatment.

Several methods have been developed to examine the baroreflex physiology in humans (Parati et al., 2000). In general, the baroreflex function is quantified by measuring the change in the heart rate and/or muscle sympathetic nerve activity in response to provoked and/or spontaneous changes in the blood pressure. However, the blood pressure responses to baroreflex perturbations cannot be evaluated using these approaches. The variable-pressure neck chamber technique offers a unique solution to this problem and exhibits several advantages including, but not limited to, the precise control of the rate, intensity, timing, and duration of the pressure stimulus, and realization of the selective activation or deactivation of the carotid baroreceptors by applying a measurable positive or negative pneumatic pressure to the neck region (Eckberg, 1977a; Fadel et al., 2003). Although these advantages highlight the utility of the variable-pressure neck chamber in assessing the carotid baroreflex function in human experimental investigations, the existing neck chamber devices produce a loud noise during their operation, similar to the suction sound of vacuum cleaners (Ludbrook et al., 1977; Cooper and Hainsworth, 2001). Moreover, noise annoyance is often associated with acute and chronic alterations in the cardiovascular system (Münzel et al., 2018), and hence, this confounding influence should be avoided in human cardiovascular physiology examinations. Consequently, it is essential to develop a noiseless variable-pressure neck chamber device.

Considering this background, this study was aimed at developing a noiseless neck suction chamber device (NCD) that could stimulate the baroreceptors located in the carotid sinus in humans. Furthermore, the neck chamber was developing using a 3D printer as a novel technique, with a focus on enhancing the subject comfort and pressure sealing.

## Materials and Methods

### Ethics

All the study procedures were approved by the institutional research ethics committee (CAAE: 26228819400005103) in accordance with the Declaration of Helsinki. Written informed consent was obtained from the individuals for the publication of any potentially identifiable images or data included in this article.

### Subjects

Data were collected from six healthy, young, asymptomatic participants (five men; mean ± SD; age: 32 ± 6 year; height: 1.7 ± 0.1 m; weight: 69 ± 7 kg; and body mass index: 24 ± 2 kg/m^2^), who were normotensive, nonsmoking, in sinus rhythm, free of known cardiovascular or metabolic diseases, and not consuming any acute or chronic medications. Before each study visit, the participants abstained from caffeine, alcohol, and strenuous exercise for 12–24 h.

### Neck Chamber

The neck chamber device was built based on the principles described by Eckberg et al. (1975) and Raine and Cable (1999). The proposed device involved mechanical and electronic components controlled by software through a portable display. The device was designed to apply controllable pressure within a given range (from 0 to −80 mmHg) to stimulate the carotid baroreceptor.

**Figure 1:**
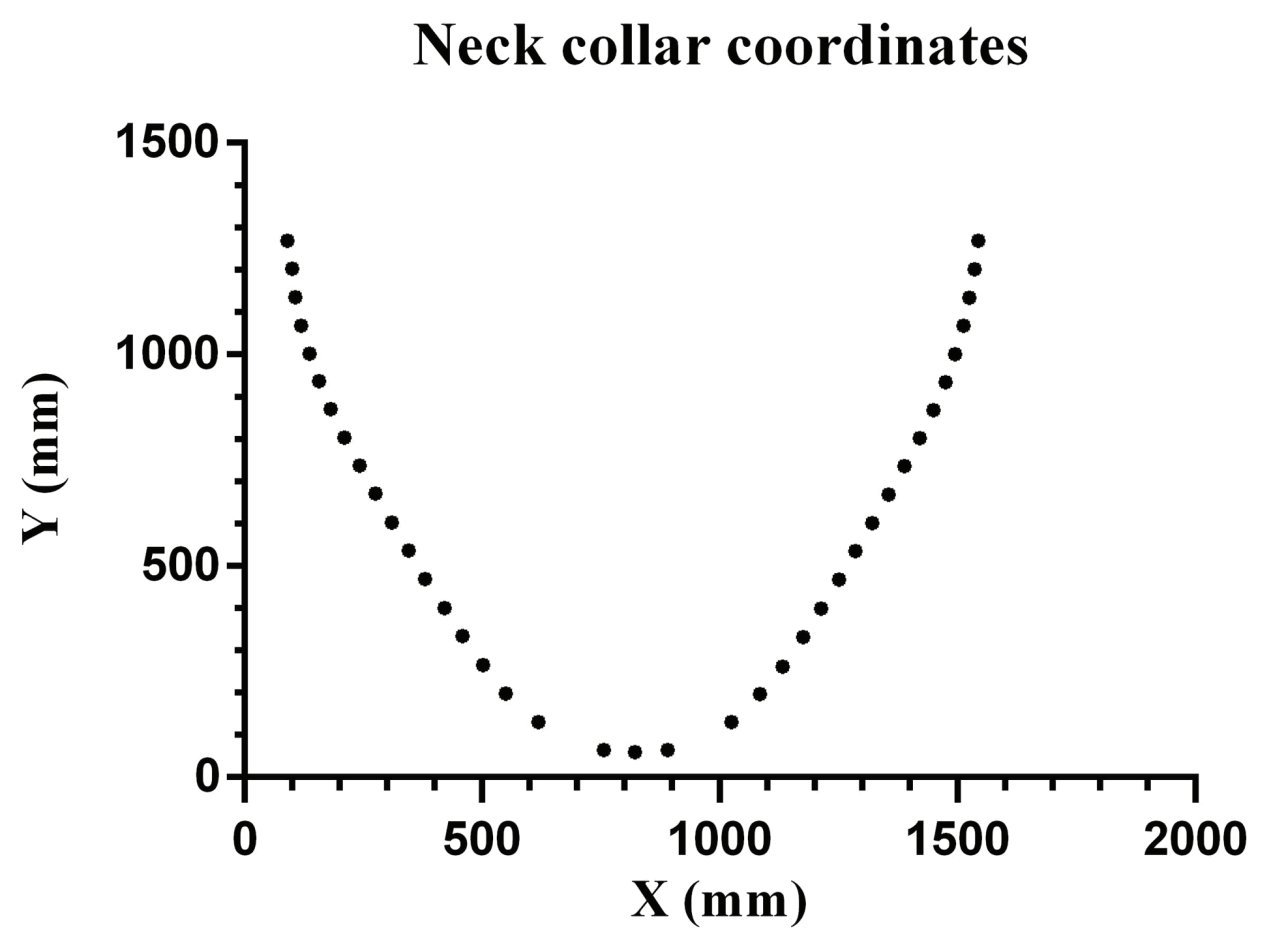
Points representing the coordinates to model a medium-sized human neck.

The development of the neck collar was a very challenging step. After several “trial-and-error” experiences, we realized that the mandibular shape is a key element affecting chamber fit, independent of weight, height, and/or musculature. Our proposed equation is based on the idea of a V-shaped mandible and it was derived from six subjects. The average coordinates were obtained, representing the average natural curves of the specific body region. Three different sizes were designed to ensure that the device could fit people with different body structures. The coordinates were uploaded to software (Inventor Professional 2020, Autodesk, California, United States) to design the best curve to fit each size. Figure 1 shows the coordinates of the median size neck. The model with nine degrees of the curve (MATLAB R2018a, MathWorks, Massachusetts, United States) can be expressed as


$$\begin{array}{l} f(x)=\;\;-3.4e-26\;\;*\;x9\;\;+\;\;1.9e\mathrm{-21}\;\;*\;\;x8+\;\;3.9e\mathrm{-17}\;\;*\;\;x7+\;\;4.4e\mathrm{-13}\;\\ \;\;*\;\;x6-\;\;2.9e\mathrm{-09}\;\;*\;\;x5+\;\;1.285e\mathrm{-05}\;\;*\;\;x4-\;\;0.035\;\;*\;\;x3\\ \;+\;\;62.78\;\;*\;\;x2-\;\;6.27e\;+\;\mathrm{04}\;\;*\;\;x+\;\;2.73e\;+\;\mathrm{07}.\; \end{array}$$

**Figure 2:**
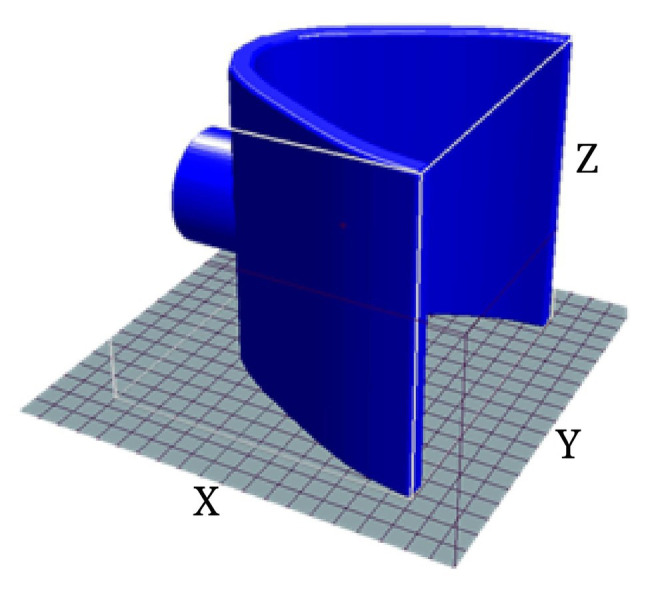
3D design of the neck chamber, according to the coordinates.

The neck chamber (Figure 2) was printed using a 3D printer (i3, RepRap, Bath University, United Kingdom), using a flexible filament (TPU 95A, National 3D, São Paulo, Brazil).

### Mechanical

**Figure 3:**
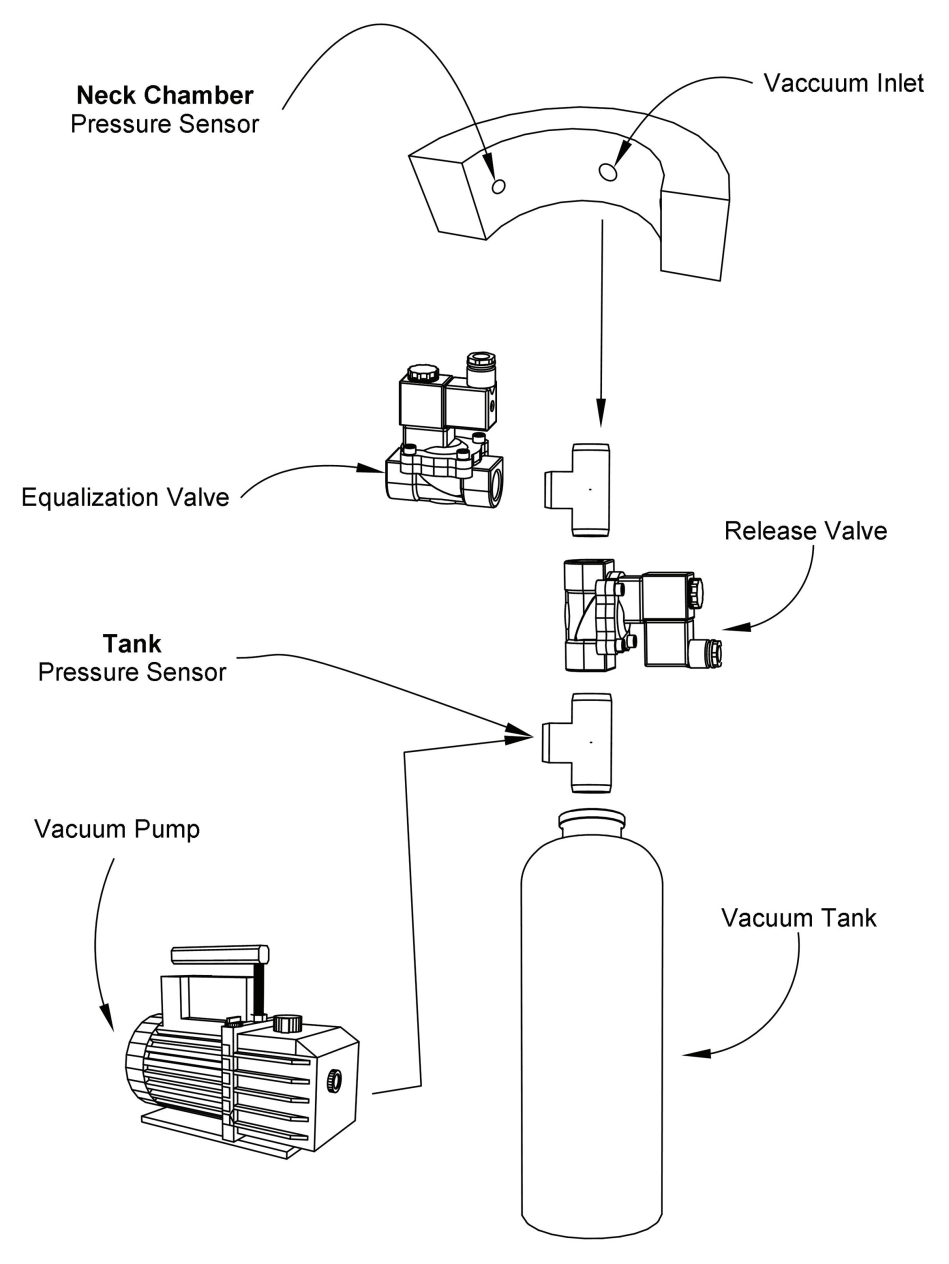
Diagram of the mechanical part of the neck chamber device.

**Figure 4:**
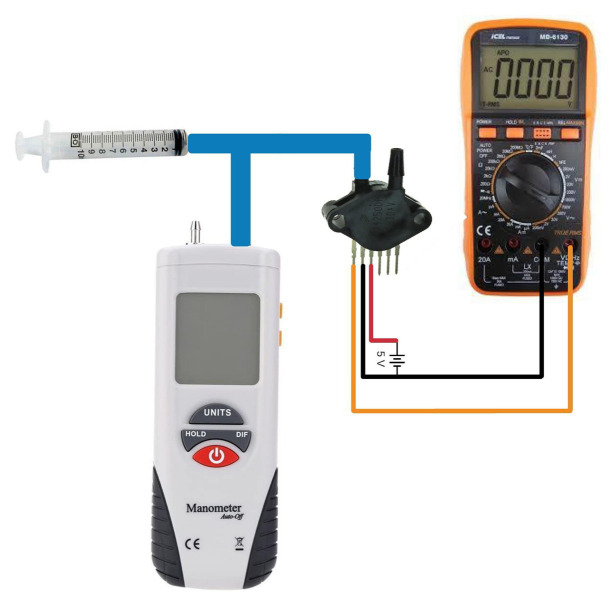
Method of calibrating the pressure sensors. A syringe is used to vary the pressure of the sensor. The multimeter measures the voltage values at the sensor’s output.

The functional components in the mechanical part include a silent vacuum pump (Airmed D400 220 V/60 Hz, Sao Bernardo do Campo, Brazil) combined with a vacuum tank (10 L, Gasnag, São Paulo, Brazil) to ensure that the pressure can be varied sharply and rapidly. Owing to the vacuum tank, the pump does not need to operate during the entire test, thereby leading to a quiet operation. As shown in Figure 3, two valves (Tcontrol, AC220V2L3505, São Paulo, Brazil) are used. The release valve opens to provide negative pressure, and after this suction operation, the equalization valve opens, returning the pressure in the neck chamber to the ambient pressure. Two pressure sensors (NXP Semiconductor, MXP5010dp, Eindhoven, Netherlands) are installed, one in the tank and the other in the neck chamber. All the sensors were calibrated using an external pressure monitor (HT-1890, Rise, China) and a multimeter (MD-6130, ICEL, Manaus, Brazil). Through five calibration points, the respective voltage values were found for each pressure value manually adjusted by the syringe, according to the calibration scheme shown in Figure 4. All the mechanical processes are computer-controlled, as described in the subsequent section.

Tests were performed to evaluate the noise produced by the proposed system. The REW software (Room EQ Wizard V15.9, John Mulcahy, United States) was used to analyze the sound pressure level (SPL). The SPL of the mechanical system was measured using a USB microphone (Kolke, KPI-271, Espírito Santo, Brazil) placed 1.5 m from the system.

### Electronic

**Figure 5:**
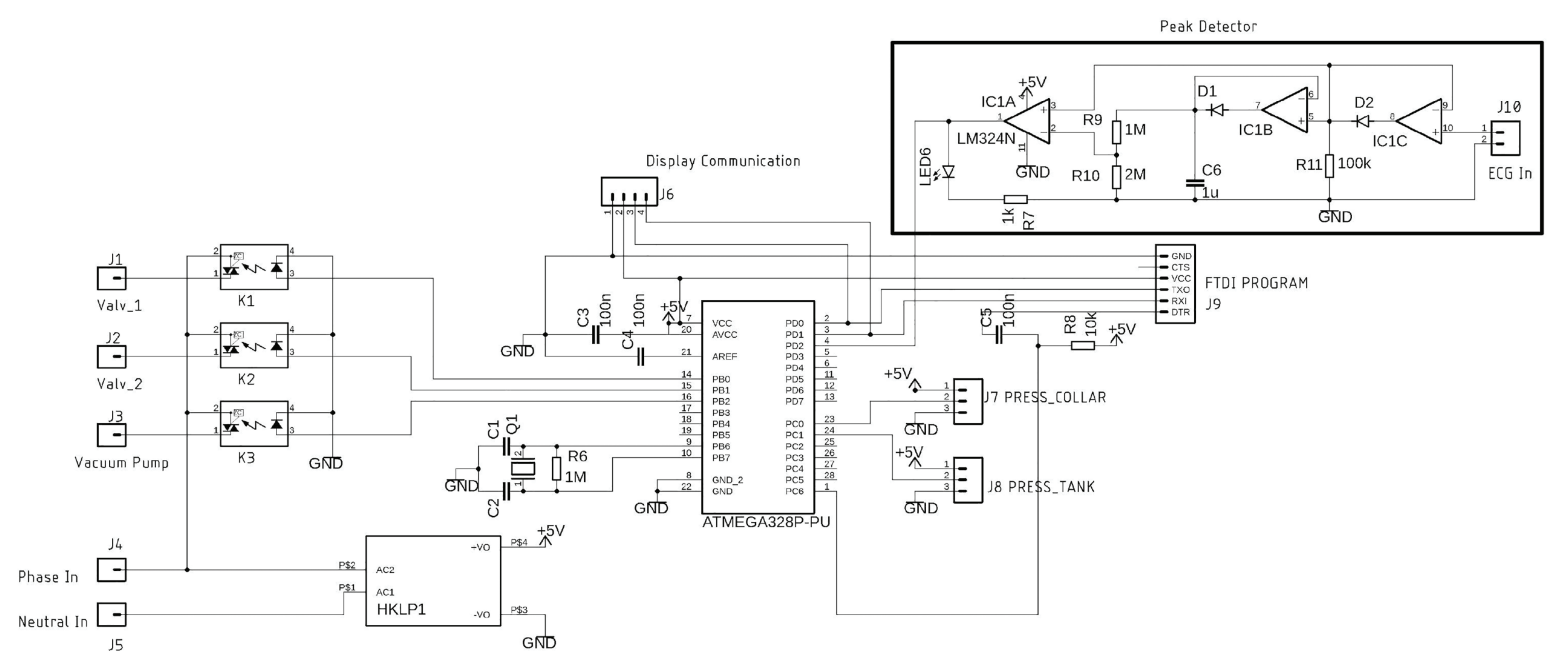
Schematic of the electronics of the neck chamber device, including the R-peak detector.

As shown in Figure 5, the NCD has three solid-state relays with optical coupling (k1, k2, and k3). Each relay is activated through the digital ports (PB0, PB1, and PB2) of the microcontroller ATmega328P-PU (MC). The MC, display, and relays are powered by a compact 5 V power supply (HLK-PM01, Hilink, Guangdong, China). The neck and tank pressure are continually updated at 115,200 bounds by a serial interface through the PD0 and PD1 digital ports (J7 and J8, respectively). The neck and tank pressure sensors are read the analog ports PC0 and PC1, respectively.

One ECG device (CardioMatic, MSC-6111, New York, United States) is connected to the system *via* the J10 analog MC port to provide suction when the R-wave peak occurs, directly to the peak detector circuit (Figure 5). Any ECG device can be used as long as it has an analog signal pertaining to the DII lead. The analog ECG signal is rectified in a half-wave using an operational amplifier (LM324). In general, the ECG signal can have different amplitudes, and each peak can be appropriately detected by the diodes that only lead to positive signals in direct polarization. In the last operational amplifier (IC1A), pin 3 has an original signal input, and pin 2 receives an attenuated signal through the voltage divider (R9 and R10). If the electric tension level in pin 3 is higher than that in pin 2, the output changes to high, thereby detecting the peak. The software continuously stores the duration of six consecutive RR intervals in the MC.

**Figure 6:**
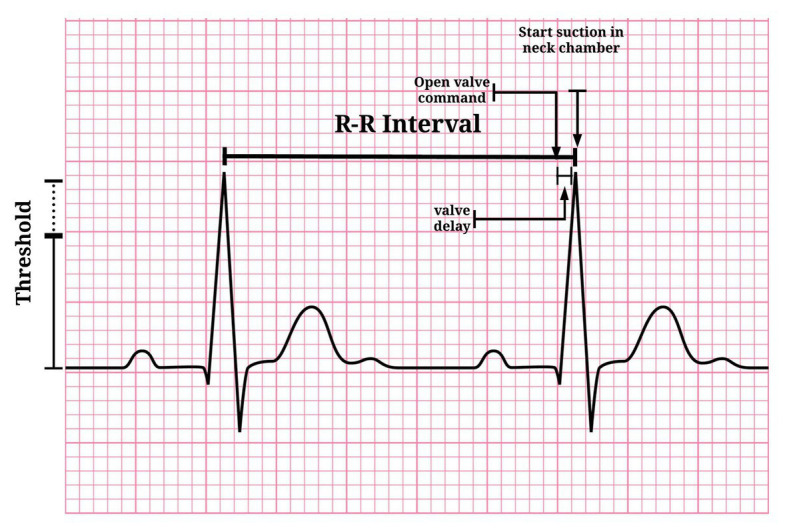
Neck suction start point is advancing due to valve delay.

**Figure 7:**
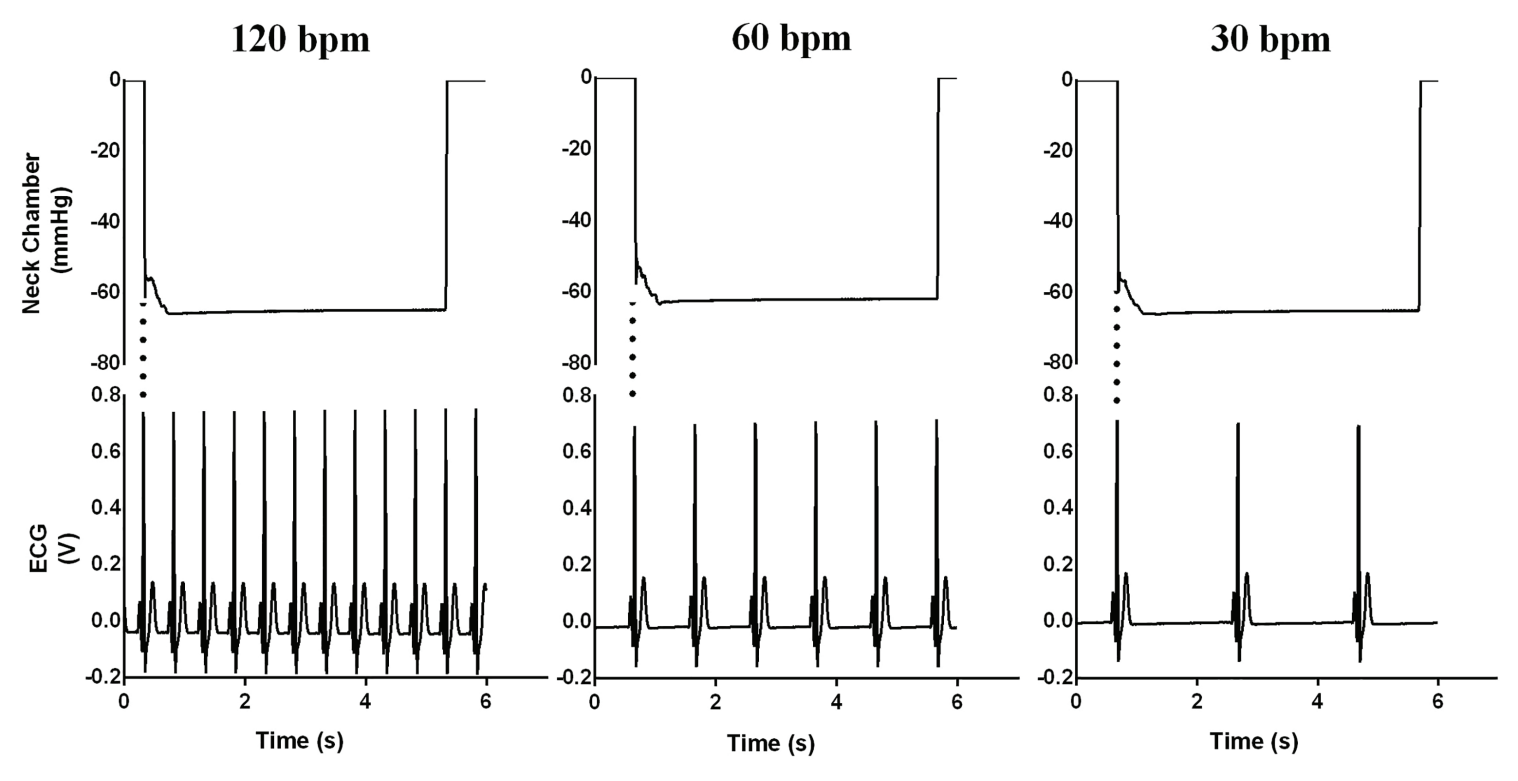
Evaluation of ECG trigger at frequencies of 30, 60, and 120 bpm.

When the operator presses the “start” button, the MC checks whether there is a stable condition (i.e., variability less than 5% among six RR intervals). If it is stable, the exact moment to trigger the valves will be calculated based on the predicted RR interval duration (based on the stability of the previous six RR intervals), subtracted by the delay of the valves (~27 ms). Therefore, the valves are opened in ~27 ms before the next R-wave peak. This algorithm ensures that the suction is applied close to the R-wave peak. Figure 6 shows when neck suction starts in the QRS complex, considering the delay of the valves. The triggering system performance was evaluated using a variable ECG simulator (30, 60, and 120 bpm, Figure 7).

**Figure 8:**
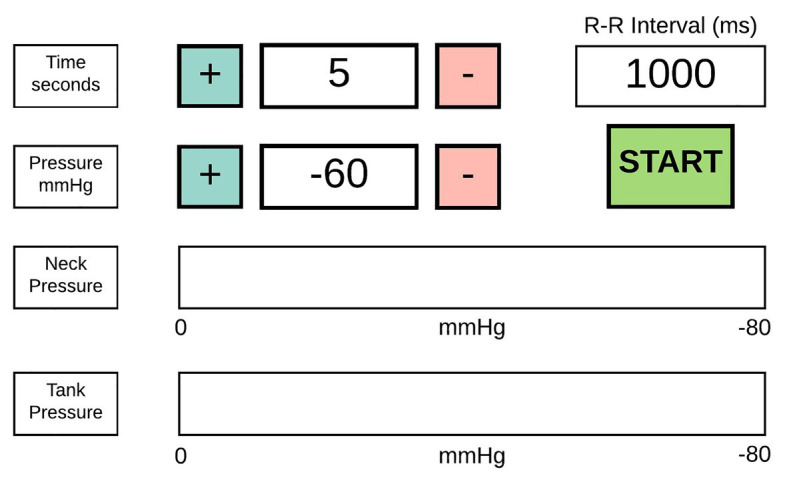
Graphical interface of the neck chamber device.

A user interface (Figure 8) was developed for a display (NX8048K070_011C, Nextion, Shenzhen, China). The display was touch-sensitive and capacitive and could ensure real-time serial communication (115,000 bps) with the MC. The time and pressure indicate the duration and intensity of the suction, respectively. Values such as the neck and tank pressure and R-R interval were transmitted to the display continuously. This interface could adjust the pressure (ranging from 0 to −80 mmHg) and time stimulus (0–60 s).

The neck suction was applied at <50 ms after the R-wave peak (Credeur et al., 2014; Barbosa et al., 2016; Ogoh et al., 2018). This timing (i.e., <50 ms) allows synchronicity between the pressure wave from the cardiac cycle (i.e., systole – the phase of the cardiac cycle when blood is being pumped out of the heart, which begins approximately 50 ms after the R-wave peak) and the neck suction pressure pulse at the carotid sinus (Eckberg, 1977b).

### Protocol

After an explanation of the protocol to the participants, written informed consent was obtained, then a carotid ultrasound scanning was performed in all participants in order to check for the presence of atherosclerotic plaques. Then, the system operator set the protocol on the display. First, the operator set the duration of the stimulus (seconds) and suction intensity (mmHg). To maintain the selectivity of the neck suction to isolate the carotid-baroreflex-mediated responses, the stimulus was required to be brief to avoid any adaptation of the carotid baroreceptors or counteraction from the extracarotid baroreceptors (Fadel et al., 2003). The duration adopted in this study was 5 s, as it is the optimum duration for the stimulation to obtain peak heart rate and blood pressure responses (Eckberg, 1977a,b; Fadel et al., 2003).

The participants remained seated at least for 5 min to stabilize the cardiovascular components (Vianna et al., 2018). The respiratory movements were monitored using a belt placed around the abdomen (MLT 1132 Piezo Respiratory Belt Transducer, ADInstruments, Sydney, Australia). The blood pressure was continuously measured (beat-to-beat) through a photoplethysmography device (Human NIBP Controller, ADInstruments) placed on the middle finger of the non-dominant hand of the participants. All the signals were collected through an integrator (PowerLab 16/35, ADInstruments), at a sampling rate of 1 kHz.

After stabilizing the signals, the neck chamber was comfortably positioned to involve the anterior two-thirds of the neck (Querry et al., 2001; Krnjajic et al., 2016). Under resting conditions, each pressure stimulus was delivered to the carotid sinus during a 10–15 s breath-hold at the end-expiration phase to minimize the respiratory-related modulation of the heart rate and mean arterial pressure (Eckberg et al., 1980).

Four to five trials of neck suction were performed with a minimum of 45 s of recovery allotted between trials to allow all physiological variables to return to pre-stimulus values (i.e., three cardiac cycles average immediately preceding neck suction). Carotid baroreflex-mediated changes in cardiovascular variables were calculated from the pre-stimulus values and plotted on a beat-to-beat scale. The responses were calculated through the lowest value (nadir) obtained during neck suction and the respective pre-stimulus baseline. Changes in all cardiovascular variables in response to individual trials neck suction were averaged for each subject and then combined to provide a group mean (A. Kim et al., 2011). Of note, since the present system only operates to assess cardiovascular changes to neck suction, we were unable to model our data using the logistic model to determine a baroreflex sigmoidal curve fit.

All the data were presented as means. All variables showed normal distribution in the Shapiro–Wilk test. One-way repeated-measures ANOVA was used to compare pre-stimulus, neck suction, and post-stimulus. The Greenhouse–Geisser correction was used to adjust ANOVA results whenever sphericity was violated in the Mauchly test. The Bonferroni *post hoc* was used when significant *F* values were found. All analyses were two-tailed, and statistical significance was accepted for *p* < 0.05. Statistical analyses were performed using Statistical Package for the Social Sciences, version 20.0 for Windows (SPSS, Chicago, IL).

## Results

**Figure 9:**
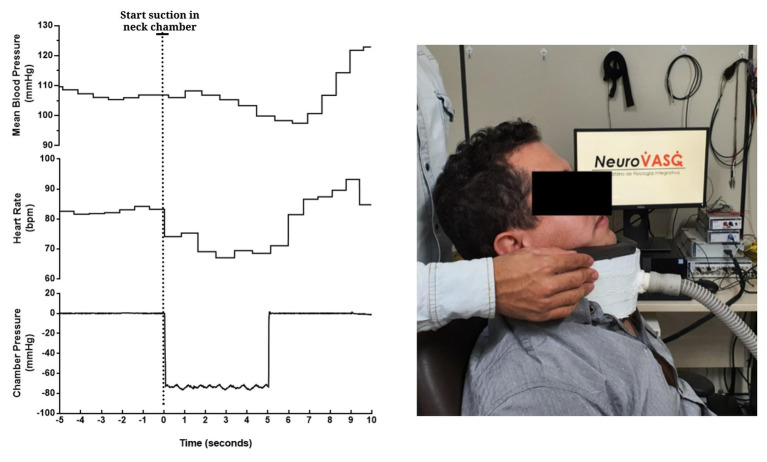
Beat-to-beat tracings from a young man showing the temporal pattern of mean blood pressure (top), heart rate (mid), and chamber pressure (bottom) during acute carotid baroreceptor hypertensive stimuli (i.e., neck suction, −60 mmHg). Neck chamber placed on a participant (right panel).

**Figure 10:**
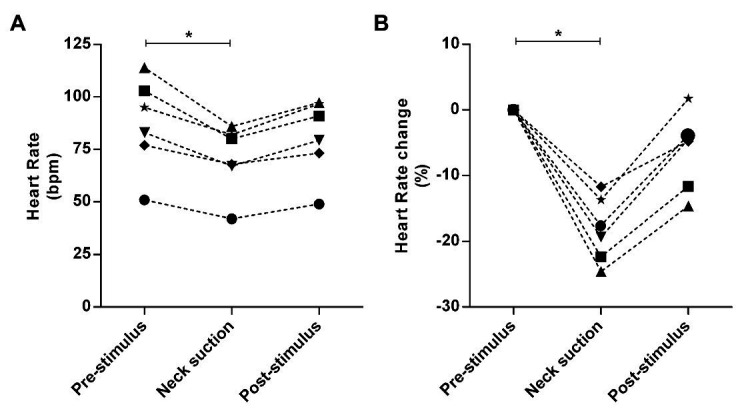
Absolute and relative responses are shown in panels A, and B, respectively. The responses were calculated through the lowest value (nadir) obtained during neck suction and the respective pre-stimulus baseline, i.e., 3-bpm average immediately preceding neck suction. *p < 0.01.

In all the trials of a representative subject, consistent reflex bradycardia (−10 ± 2 bpm) and depressor response (−15 ± 4 mmHg) to the neck suction were observed (Figure 9), and these responses are similar to those reported in the literature (Ernsting and Parry, 1957; Huang et al., 2016). The absolute (pre-stimulus: 87 ± 9 bpm; neck suction: 71 ± 7 bpm; post-stimulus: 81 ± 8 bpm; *p* < 0.01; Figure 10A) and relative (pre-stimulus: 0%; neck suction: −18 ± 2%; post-stimulus: −6 ± 2%; *p* < 0.01; Figure 10B) heart rate responses to neck suction were significantly different compared to pre-stimulus values.

The ECG trigger had a delay of less than 50 ms in all attempts, as shown in Figure 7. Four attempts of neck suction trials (−60 mmHg) were performed for each participant. The rate of pressure change was ~3,000 mmHg/s.

**Figure 11:**
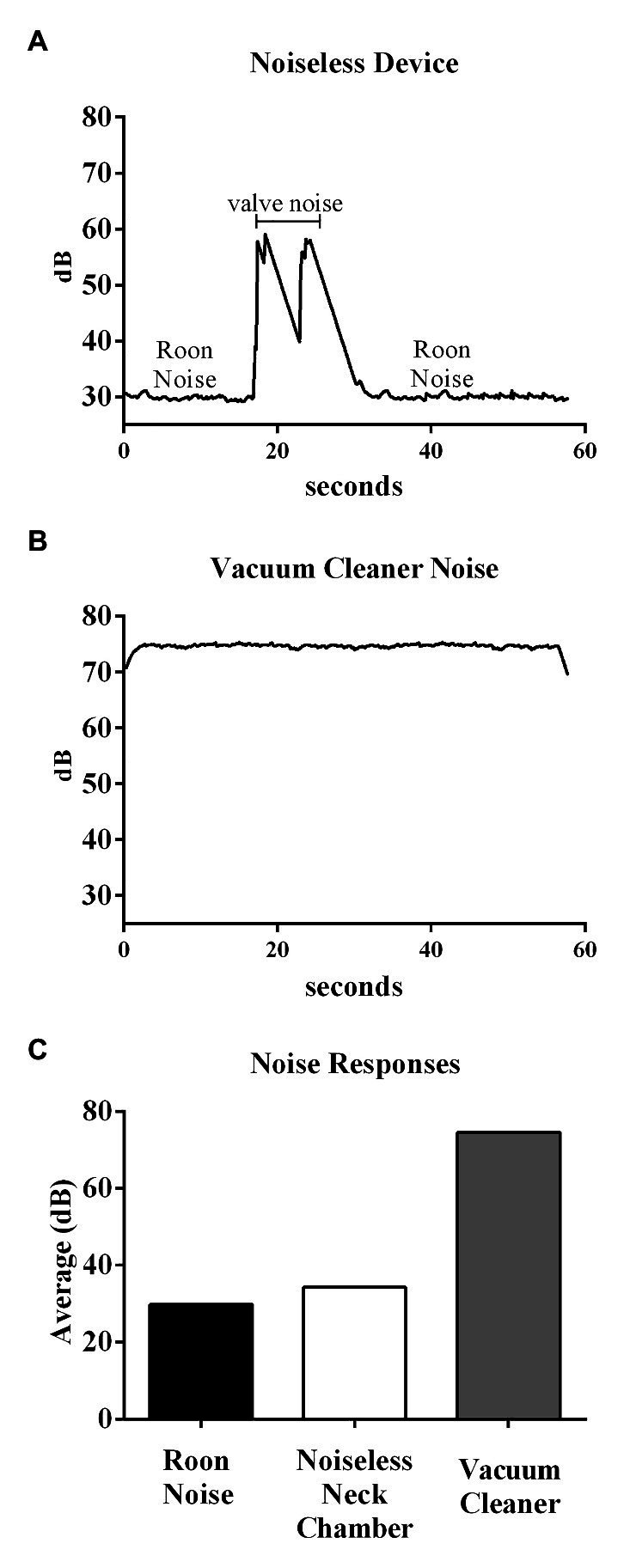
Top and middle panels show the original recording of the noise from the noiseless device and Vacuum cleaner-based system, respectively. Bottom panel shows the average noise responses within different environments.

The proposed device had an average SPL of 34.3 dB, as shown in Figure 11, considerably smaller than that of the vacuum-cleaner-based device (74.6 dB). Note that, the SPL of a silent room is usually approximately 30–34 dB.

After a five-point calibration of the sensors, the equation was obtained, which shows linearity between pressure and electrical voltage:


V=−0.037p+0.798

Where *V* is the voltage at the output pin of the pressure sensor, and *p* is the pressure (mmHg).

## Discussion

Several trials were performed on the participants to examine the efficacy of the proposed neck suction technique in terms of generating the reflex responses, in a similar manner as those reported in previous studies. Before each trial, the system operator set the values of pressure (−60 mmHg) and duration (5 s) for the carotid stimulus. In this study, a noiseless neck suction device was developed to activate the carotid baroreceptors in humans. In accordance with the main objective of the study, the developed device can perform noiselessly, thereby enhancing the participant comfort during the execution of the protocol and providing a suitable environment to examine the baroreflex physiology. The uniqueness of the proposed system pertains to the use of a vacuum tank in which the vacuum is stored, which enables the realization of a rapid and silent pressure change inside the neck collar whenever the neck suction is implemented. In comparison, the noise level of the conventional system used in the existing studies is significantly higher as the motor pump must be operated (vacuum cleaner motor) to provide a continuous vacuum source (Cooper and Hainsworth, 2001). Alternatively, a previous fMRI study has placed the neck suction engine outside the examination room (Makovac et al., 2018) aiming to reduce the noise, although no data were presented in terms of noise during its operation. In contrast, the advantages of the present system lie in the detailed open-source descriptions of the hardware, and electronics, and the development of a “silent” and reasonably portable neck suction device.

In the evaluation, the vacuum reservoir was noted to considerably influence the release and stability of the vacuum. Although the collar was pressurized instantaneously, extremely small fluctuations occurred on the negative plateau, although these were considered to be irrelevant taking into account their possible physiological impact on the carotid baroreflex-mediated reflex responses. The timing of the suction onset was satisfactory, as the peak of the R-wave was observed to exactly coincide with the beginning of the collar pressurization. Nevertheless, it is important to state that our proposed system works only in steady-state conditions. As such, the present algorithm/system does not work in the presence of cardiac arrhythmias or during the transition from rest to exercise (exercise onset). For carotid baroreflex testing in such conditions, the algorithm could be modified. Future studies should investigate its feasibility. In addition to the tank, the role of the malleable neck chamber was significant, as the flexible material allowed a better adaptation to the neck around the anterior two-thirds of the neck and enhanced the participant comfort. Furthermore, the applied silicone coating helped avoid any vacuum leakage.

To activate the carotid baroreceptors through the neck chamber technique, certain parameters must be suitably set. For example, the rate of pressure change in the neck chamber must be extremely high to provide an uniform stimulation throughout the 5 s suction window. Accordingly, in this study, the rate of change was set higher than ~3,000 mmHg/s, which is considered to be sufficient to extend the baroreceptors located in the carotid sinus (Eckberg, 1977a,b).

In the proposed device, the analog circuit for R-wave detection is an essential component because the circuit adapts the gain of the signal to the analog port of the MC, to supply a constant voltage. It is expected that the systems pertaining to a vacuum cleaner motor likely cannot adequately control the amount of suction compared to in the proposed device; nevertheless, further work is necessary to validate this aspect.

The neck collar could generate reproducible responses in the case of all the participants, and consistent carotid baroreflex-mediated responses to neck suction were observed, similar to those reported in the previous publications. The key advantage of the proposed NCD pertained to its quiet operation. The noise produced during a 1 min trial was slightly more than 30 dB (equivalent to that of a whisper); in contrast, the vacuum-cleaner-based system produces a noise of 70–80 dB (equivalent to a passenger car or a telephone ringing). Although we did not test how the acute exposure to noise could directly affect the reflex responses to the neck suction, it has been reported that intermittent exposure of monkeys to noise (85 dB) for 9 months significantly increased the blood pressure by 30 mmHg, even though the auditory system was not notably influenced (Münzel et al., 2018). Furthermore, according to the United States Environmental Protection Agency (1974), the noise levels in hospital environments should not exceed 45 and 35 dB during the day and night, respectively. The World Health Organization recommends a sound level of 30–40 dB in internal hospital environments (Berglund et al., 2000). The proposed neck chamber device, with its unique low noise operation, can thus be widely applied in hospital environments.

## Conclusion

In the tests using the proposed device, consistent blood pressure and heart rate responses to carotid baroreflex hypertensive stimuli were observed, consistent with the results of previous studies involving the use of neck collar devices. Moreover, the proposed device could realize noiseless operation, thereby providing a *sine qua non* environment for the baroreflex assessment in human physiology laboratories.

## Data Availabiltity Statement

The datasets and/or codes generated during and/or analyzed during the current study are available from the corresponding author on reasonable request.

## Ethics Statement

The studies involving human participants were reviewed and approved by Faculdade de Ciências Médicas e da Saúde de Juiz de Fora/FCMS/FJ/SUPREMA – CAAE: 26228819400005103. The patients/participants provided their written informed consent to participate in this study.

## Author Contributions

AP, LV, and JC wrote the manuscript and performed the data analysis. All authors contributed to the article and approved the submitted version.

### Conflict of Interest

The authors declare that the research was conducted in the absence of any commercial or financial relationships that could be construed as a potential conflict of interest.
