# Erratum: Noiseless variable-pressure neck chamber device to assess the carotid baroreflex function

**DOI:** 10.3389/fphys.2023.1244387

**Published:** 2023-07-12

**Authors:** Frontiers Production Office

**Affiliations:** Frontiers Media SA, Lausanne, Switzerland

**Keywords:** blood pressure, baroreflex, neck suction, hypertensive stimulus, neck collar

Due to a production error, the wrong **DOI** was included in the XML metadata.

The publisher apologizes for this mistake. The original version of this article has been updated.

